# Combining Liquid Chromatography and Cryogenic IR Spectroscopy in Real Time for the Analysis of Oligosaccharides

**DOI:** 10.1021/acs.analchem.3c03578

**Published:** 2024-01-11

**Authors:** Ali H. Abikhodr, Stephan Warnke, Ahmed Ben Faleh, Thomas R. Rizzo

**Affiliations:** Laboratoire de Chimie Physique Moléculaire, École Polytechnique Fédérale de Lausanne, EPFL SB ISIC LCPM, Station 6, CH-1015 Lausanne, Switzerland

## Abstract

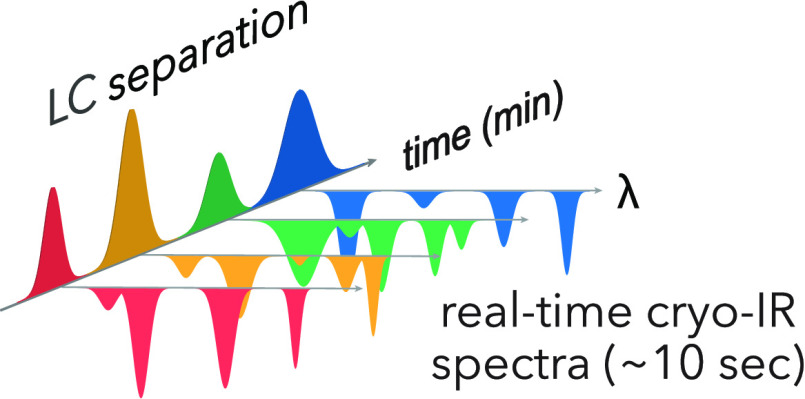

While the combination of liquid chromatography (LC) and mass spectrometry (MS) serves as a robust approach for oligosaccharide analysis, it has difficulty distinguishing the smallest differences between isomers. The integration of infrared (IR) spectroscopy within a mass spectrometer as an additional analytical dimension can effectively address this limitation by providing a molecular fingerprint that is unique to each isomer. However, the direct interfacing of LC-MS with IR spectroscopy presents a technical challenge arising from the mismatch in the operational time scale of each method. In previous studies, this temporal incompatibility was mitigated by employing strategies designed to slow down or broaden the LC elution peaks of interest, but this workaround is applicable only for a few species at a time, necessitating multiple LC runs for comprehensive analysis. In the current work, we directly couple LC with cryogenic IR spectroscopy by acquiring a spectrum in as little as 10 s. This allows us to generate an orthogonal data dimension for molecular identification in the same amount of time that it normally takes for LC analysis. We successfully demonstrate this approach on a commercially available human milk oligosaccharide product, acquiring spectral information on the eluting peaks in real time and using it to identify both the specified constituents and nonspecified product impurities.

## Introduction

The analysis of oligosaccharides is of paramount importance, given their diverse roles in biological systems. These carbohydrate polymers are ubiquitous in nature and are integral to a variety of physiological processes. In human health, they play essential roles in immune response modulation,^1−3^ cell–cell communication,^4,5^ and as prebiotics in the gut microbiome, influencing digestion and overall gut health.^6−8^ Aberrations in glycosylation patterns have been associated with numerous diseases, including cancer and neurodegenerative disorders.^9−11^ Despite their importance, the analysis of glycans is significantly hindered by multiple sources of isomeric complexity. Many of the monosaccharide building blocks are isomeric, often differing in the stereochemistry of a single carbon atom. Multiple OH groups of a single monosaccharide provide a variety of linkage points via glycosidic bonds, each of which can be in the α or β configuration, resulting in anomers and regioisomers as well as allowing the formation of branched structures. Moreover, the multiplicity of OH groups that can be functionalized leads to various positional isomers. Distinguishing all possible isomeric forms for a glycan of a given mass presents a formidable analytical challenge.

Traditional mass spectrometry-based approaches for glycan analysis typically rely on a prior isomer separation step using liquid chromatography (LC),^12,13^ ion mobility spectrometry (IMS),^14,15^ or capillary electrophoresis (CE).^16,17^ While the coupling of these techniques allows for the separation of the components of complex mixtures, the challenge is in assigning their precise isomeric forms. The currently preferred strategy for analysis and identification often integrates these separation techniques with tandem MS, which relies on the observation of isomer-specific fragments.^18−22^ However, the capacity to discriminate between subtly different isomers remains a challenge because their fragmentation patterns often exhibit only small changes in relative intensities. Such differences are often not universal and can depend on instrument conditions and platforms.

To address this issue, a number of groups^23−28^ have explored the addition of a new analytical dimension—that of an infrared (IR) spectrum, which provides a unique molecular fingerprint for each molecule that can distinguish isomers. Because of the richness of information contained in an IR spectrum, particularly when performed at cryogenic temperatures, an analyte can be confidently identified when matched with a database spectrum without the need for database entries for all plausible isomeric forms. We have previously demonstrated the combination of cryo-IR spectroscopy with IMS-MS.^29,30^ Because of the ubiquity of LC workflows and their importance for minimizing ion suppression effects in complex mixtures, the combination of LC with IR spectroscopy would be an important addition to the oligosaccharide analysis toolbox. However, the integration of IR spectroscopy with LC-MS has been fraught with technical challenges, predominantly due to the disparity in operational time scales between the methods. In previous implementations, recording an IR spectrum of ions inside a mass spectrometer required tens of minutes, which is much greater than the widths of peaks eluting from a liquid chromatograph. To deal with this temporal incompatibility, past methods have either extended the elution time of LC peaks of interest or used online fractionation and reinjection at lower flow rates.^31,32^ While the latter approach is promising, it is limited in its applicability as it necessitates multiple LC analyses, which can be impractical for large sample sets.

Building upon our previous brief report,^33^ we describe here an approach that directly couples LC-MS with IR spectroscopy by reducing IR spectral data acquisition time to as little as 10 s, which allows obtaining valuable molecular information while maintaining the high throughput of LC-MS. We demonstrate this technique by applying it to a commercially available nutritional supplement for children that is specified to contain five human milk oligosaccharides. Within a single LC analysis, we used IR spectra to identify the five expected commercial glycans in addition to impurities not specified by the supplier.

## Experimental Methods

### Sample Preparation

A commercial nutritional supplement containing five human milk oligosaccharides (2′-FL, LNT, LNnT, 3SL, and 6SL) for children aged 1+ was dissolved in water, as instructed on the packaging. All samples were analyzed in the sodiated charge state to avoid fucose migration, which occurs in protonated species.^34^

Prior to injection into the LC system, we diluted the sample 60-fold. Subsequently, the oligosaccharides were extracted by using a two-step process involving C18 and porous graphitic carbon (PGC) solid-phase exchange. For the C18 step, the cartridge was activated and equilibrated sequentially with 1 mL of methanol and water. Following activation, 1 mL of the sample was loaded onto the cartridge, and the flow-through was collected. Subsequently, the cartridge was subjected to three washes using 0.5 mL of water, with each wash fraction combined with the previously collected eluent. Moving onto the PGC solid-phase exchange, the PGC cartridge was activated and equilibrated sequentially with 1 mL of 60% acetonitrile/water and water. The sample was then loaded onto the cartridge and washed three times with 0.5 mL of water. Following the washing steps, the glycans were released from the cartridge using 0.3 mL of a solution consisting of 50% acetonitrile, and this process was repeated three times. Finally, 50 μL of the resulting sample was directly injected into the LC system for analysis.

### Instrumentation

**Figure 1:**
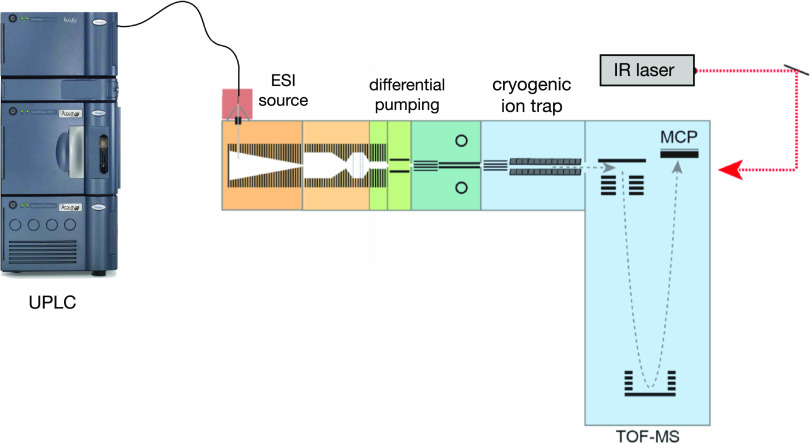
Schematic of the experimental setup.

All experiments were performed using an Acquity Premier UPLC system (Waters Corp.) coupled to our home-built instrument, shown in Figure 1.

Our home-built instrument^35^ includes a cryogenic ion trap for performing infrared (IR) messenger-tagging spectroscopy, which is connected to a time-of-flight (TOF) mass spectrometer (TOFWERK). The process begins with the molecules eluting from the PGC column, after which a separate flow of sodium acetate (10 μM) is added before the generation of ions through electrospray ionization (ESI) to facilitate the formation of sodiated species. They are then introduced into the instrument via a heated stainless steel capillary maintained at 150 °C. Initially, the ions are directed through a series of ion funnels and accumulated. Afterward, packets of ions with a duration of 1 ms are released and guided through multiple stages of differential pumping until they reach the cryogenic trap, which is maintained at a temperature of 45 K. The trapped ions are cooled by undergoing collisions with a mixture of helium and nitrogen in an 80:20 ratio, forming weakly bound clusters with N_2_. A continuous-wave mid-IR laser (IPG Photonics) is then employed to irradiate the N_2_-tagged ions for a duration of 50 ms, at which point they are released and analyzed using the TOF mass spectrometer. Upon absorption of an IR photon, the energy is redistributed among the vibrational modes of the ions, leading to dissociation of the nitrogen tag(s). The ratio between tagged and untagged ions is a measure of photon absorption of the tagged ions at a given laser wavenumber. To obtain a spectrum, this process is repeated with a repetition rate of approximately 10 Hz while the wavenumber of the laser is scanned. A spectrum over the range of 3500–3700 cm^–1^ can thereby be recorded in approximately 10 s with 100 data points over the entire spectrum.

Wavelength scanning of the IR laser is initiated by the detection of an ion signal in the TOF-MS, which is continuously triggered at a repetition rate of 10 kHz. Once the ion signal is above a certain threshold, laser scanning is automatically initiated, and the laser wavenumber is logged in the data file for each mass spectrum. A Labview program controls both the scanning of the IR frequency and the obtention of the MS, but there is no direct communication between the LC and our instrument.

### Spectral Comparison with an HMO IR Fingerprint Database

We have previously measured the infrared spectra of over 35 human milk oligosaccharides (HMOs), forming a database that allows us to directly detect the presence of these species in complex mixtures as intact molecules or as fragments of larger unknown molecules. The database spectra were recorded on the sodiated charge state of each species using N_2_ messenger tagging, and thus spectral band positions will be the same in the database and sample spectra. The structures of the molecules in our database, including monosaccharide composition and linkages, have been determined either by comparison with purchased standards or by using fragmentation-based techniques, as described in our previous work.^23,30,36−38^

We employed the Pearson correlation coefficient (PCC) to assess the similarity between the measured infrared (IR) spectra and those contained in our database. The PCC is a statistical method utilized to evaluate the linear relationship between two vector variables, and it yields a value within the range of −1 to 1. A coefficient approaching 1 indicates a robust positive correlation, while a value close to −1 suggests a strong negative correlation. A coefficient approximately equal to 0 suggests no substantial correlation between the variables. To determine the extent of similarity between our measured IR spectrum and each of the reference spectra within our database, we computed the PCC for each pair, which enables us to identify the most suitable match.

## Results and Discussion

### Identifying the Main Components

**Figure 2:**
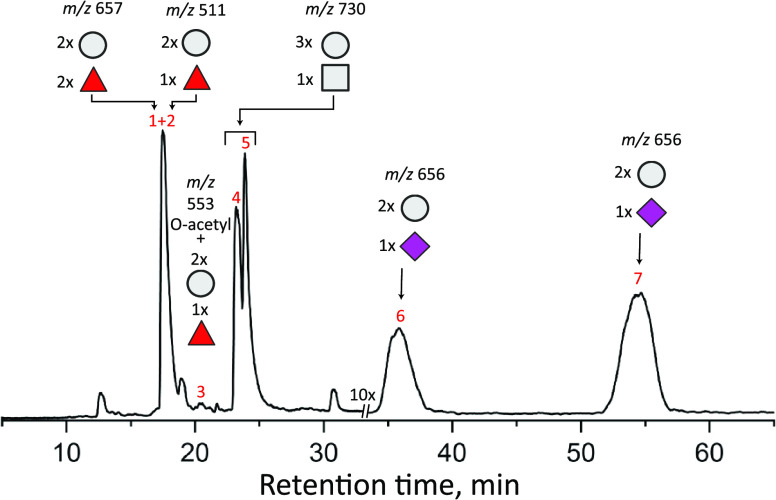
Total ion count (TIC) chromatogram of glycans eluting after PGC separation of the commercial HMO mixture. Above each peak of interest is the mass-to-charge ratio of the sodiated species and the general monosaccharide makeup, with hexose units denoted by circles, deoxyhexoses shown as triangles, and HexNAcs depicted as squares, and sialic acids are denoted as diamonds.

A chromatogram of the major elution peaks of the commercial HMO sample is shown in Figure 2. From the mass information, one can determine the monosaccharide content of the major elution peaks (see figure caption for notation), and based on the specified product constituents, one can assume that elution peak 2 is 2′-FL, peaks 4 and 5 are some combination of LNT/LNnT, and peaks 6 and 7 are that of 3′-SL and 6′-SL. Other oligosaccharides that are not mentioned on the label are also present.

**Figure 3:**
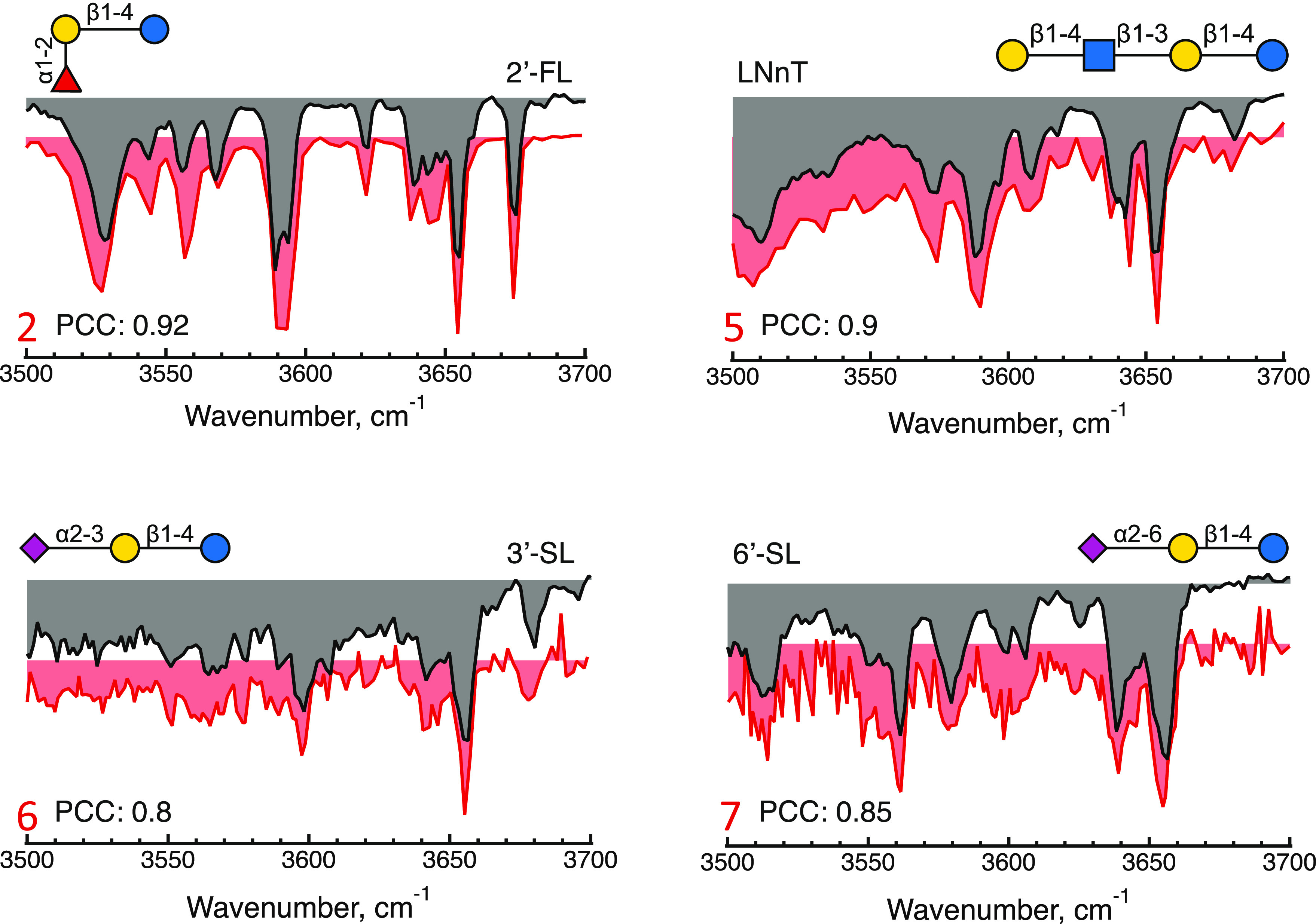
Messenger tagging IR depletion spectra of elution peaks 2, 5, 6, and 7 (in red) compared with the best identified fit. Glycan structures are drawn using the SNFG notation.^39^

To assign the retention peaks unambiguously, we acquired an IR spectrum of each of the major peaks. From comparison with the database spectra shown in gray in Figure 3, the elution peaks 2, 5, 6, and 7 can be identified as 2′-FL, LNnT, 3′-SL, and 6′-SL, respectively, with their measured spectra shown in red. Here, four of the five specified oligosaccharides were identified.

While visual inspection would be sufficient to assign these spectra correctly, determining the Pearson correlation coefficient provides an objective quantitative measure of the assignment. If, for example, we were to take the spectrum of 3′-SL and compare it to the database spectrum for 6′-SL, it would give a PCC of 0.62, considerably lower than 0.8 for the assignment of Figure 3. One expects a certain degree of correlation between spectra of isomeric HMOs because they possess the same functional groups, in this case, differing only by the orientation of the terminal sialic acid. However, if one compares the spectrum of 2′-FL with our database spectrum of 3′-FL, which differs by the attachment site of the fucose residue, it gives a PCC of 0.22. The calculated PCC thus seems to be a robust measure of the spectral assignment.

**Figure 4:**
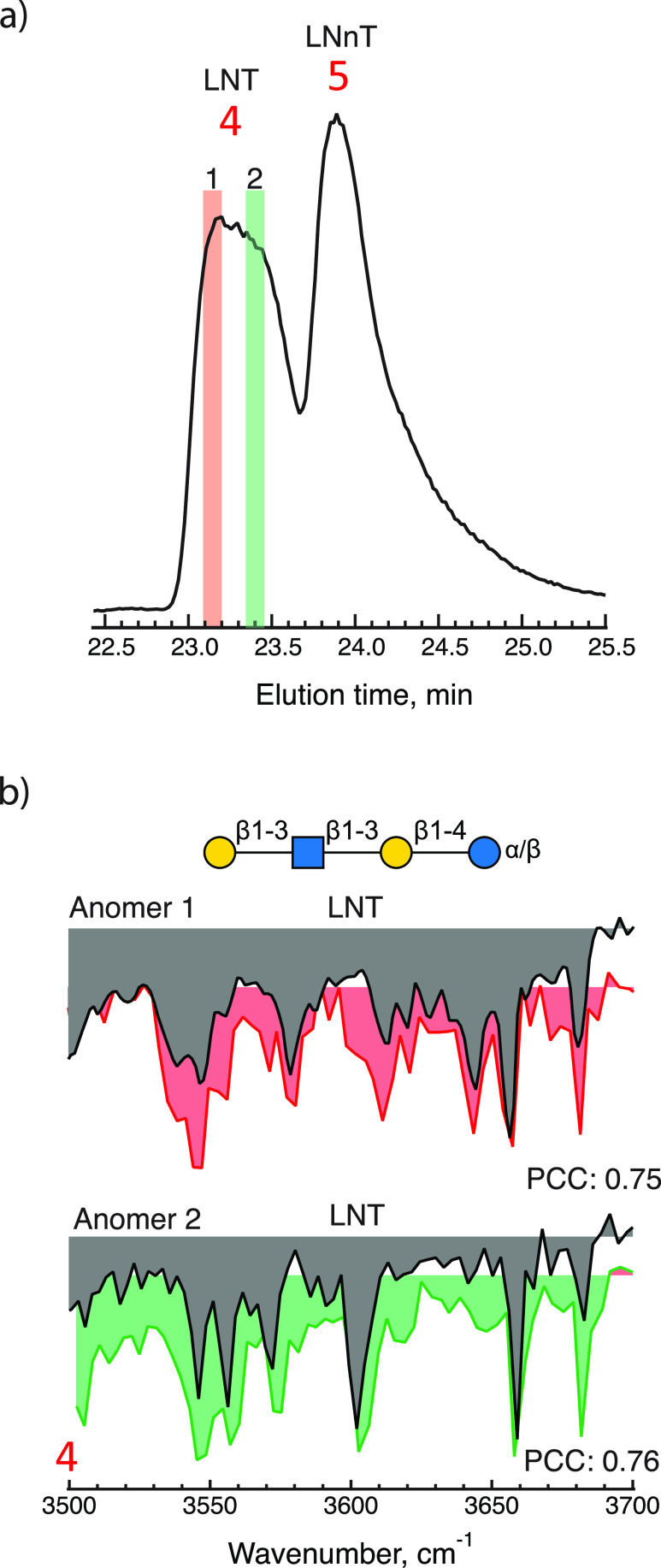
(a) Elution peak 4 on an expanded scale, highlighting the regions where messenger tagging IR spectra were taken (red and green). (b) Messenger tagging IR depletion spectra of highlighted regions compared with the best identified fit using PCC identifying the constituent of each peak.

As for the final specified component, LNT, peak 4 exhibits the correct mass, but as shown in Figure 4(a), it is broader than peak 5, which was identified as LNnT. We thus measured two IR spectra for this elution peak, one at the very beginning, highlighted in red, and one toward the end, highlighted in green. The two measured spectra shown in Figure 4(b) correspond to the α and β reducing-end anomers of the molecule LNT, as suggested in previous work.^23^ Note that comparing the spectrum of anomer 1 with the database spectrum of anomer 2 would yield a PCC of 0.37 rather than 0.75 for the assigned spectrum. This demonstrates the extreme sensitivity of the infrared spectrum to the primary structure.

The spectroscopic match of these LNT isomers further demonstrates that (1) the time scale for the mutarotation reaction between the reducing-end anomers is greater than the elution time, and (2) a PGC column has sufficient resolution to allow us to measure separate IR spectra of these isomers, which differ only by the orientation of the reducing-end hydroxy group. While this could be anticipated from our previous anomer-resolved infrared spectroscopy measurements,^40^ the ability to separate such subtly distinct isomers by liquid chromatography is noteworthy.

**Figure 5:**
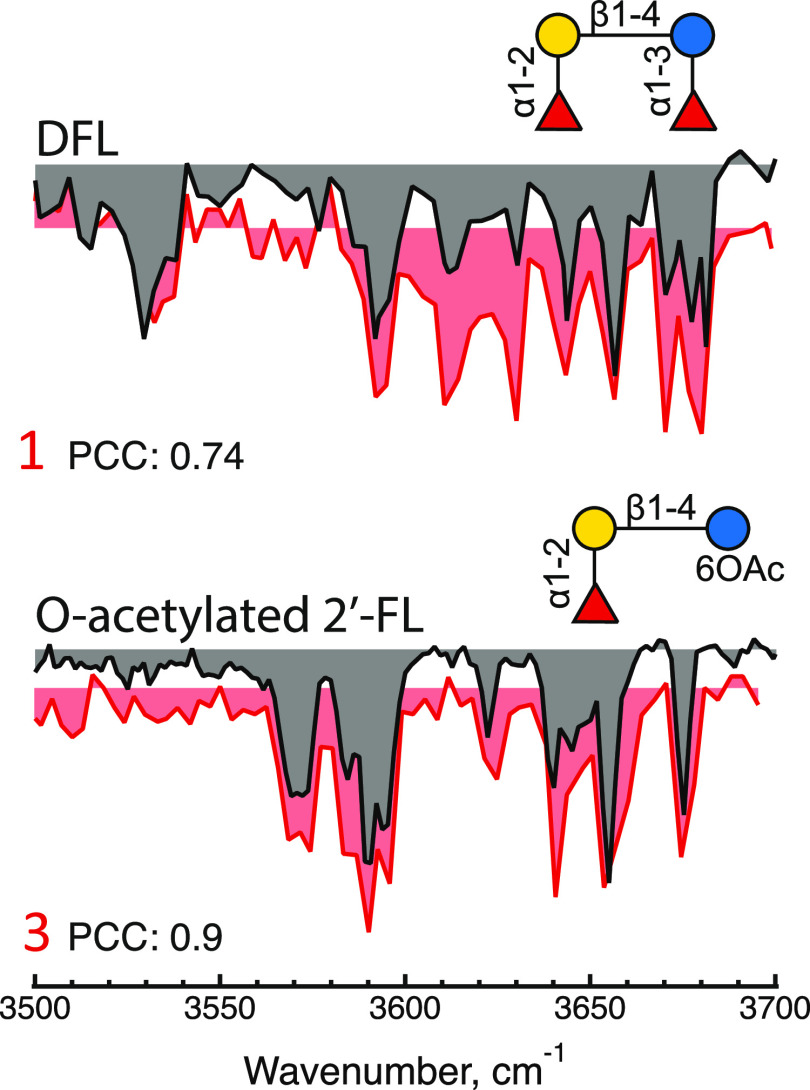
Messenger tagging IR depletion spectra of elution peaks 1 and 3 (in red) compared with the best identified fit using PCC to identify the constituent of each peak.

In addition to the HMOs that were specified components of the commercial product, two additional molecules were identified. As shown in Figure 5, by comparison with our database, we can identify peaks 1 and 3 of the chromatogram in Figure 2 as difucosyllactose (DFL) and O-acetylated 2′-fucosyllactose, with the acetylation being on the sixth position of the glucose ring. While DFL is a human milk oligosaccharide, 6-O-acetylated 2′-FL is not found in human milk, and its biological effects are unknown.

Regarding the remaining small features observed in the chromatogram of Figure 2, no corresponding matches were found within our database. These peaks could, in principle, be identified using a CID-IR method, as previously demonstrated.^30,36,38^

## Conclusions

We combined liquid chromatography with cryogenic infrared spectroscopy to facilitate the analysis of biomolecular structures. In this study, we demonstrate the utility of this approach through the analysis of a commercially available human milk oligosaccharide product. The identification of its constituent species was accomplished within a single liquid chromatography analysis using real-time cryogenic IR spectroscopy together with our expanding infrared spectroscopic database. This analytical procedure eliminated the need for calibration of elution times via internal or external standards. Moreover, we leveraged an automatic method that assigns the experimentally determined spectra by comparing them to all recorded spectra within our database to determine the most suitable match.
